# A cross‐sectional study of conflict‐handling modes among first‐year veterinary students

**DOI:** 10.1002/vro2.70023

**Published:** 2025-11-18

**Authors:** Katherine E. McCool, April A. Kedrowicz

**Affiliations:** ^1^ Small Animal Internal Medicine, Department of Clinical Sciences NC State University Raleigh North Carolina USA; ^2^ Department of Clinical Sciences NC State University Raleigh North Carolina USA

**Keywords:** communication, competency, conflict

## Abstract

**Background:**

Veterinary medical care requires communication and collaboration among members of the veterinary team. Coordinating the efforts of diverse team members can increase the potential for conflict, which can adversely impact team satisfaction and patient care when not managed productively. Awareness of conflict management styles and self‐awareness of one's own preferred mode(s) of navigating conflict are imperative to enhancing a broader understanding of conflict. The purpose of this pilot study was to characterise first‐year veterinary medical students’ preferred modes of conflict management at a single institution.

**Methods:**

Eighty‐seven students (*n* = 87) completed the Thomas‒Kilmann Conflict Mode Instrument (TKI) and descriptive statistics were performed.

**Results:**

Results revealed that accommodating (*n* = 35/87; 40.2%) was the most common preferred conflict‐handling mode, followed by avoiding (*n* = 29/87; 33.3%). Collaboration, a highly assertive and cooperative mode, was the least common preferred conflict mode (*n* = 7/87; 8.0%).

**Limitations:**

Our results are based on self‐reported data at a single point in time and may not translate to real‐world behaviour. Future research pursuits will focus on exploring how the conflict styles of veterinary students may change over time as they progress through their training and professional socialisation.

**Conclusion:**

This research suggests an opportunity for additional training in how to navigate conflict, with specific instruction on assertive and cooperative approaches to resolution. Future educational interventions related to conflict management training should be explored and additional research is needed to evaluate the most effective ways in which to incorporate these skills into veterinary medical training.

## INTRODUCTION

Communication and collaboration are core professional competencies required of veterinary medical students by both the American Association of Veterinary Medical Colleges Competency Based Veterinary Medical Education Day One Competency (Domains 5 and 6)^1^ and the Royal College of Veterinary Surgeons (RCVS) Day One Competencies (Competencies 17 and 19).^2^ In addition, there is strong evidence linking communication skills to graduate outcome success.^3^ Team effectiveness, veterinary staff members’ wellbeing, client satisfaction and patient outcomes are impacted by the veterinary team's ability to communicate effectively. The provision of veterinary medical care is dependent on effective communication and coordination of diverse team members. The diversity of perspectives, roles, hierarchical position and communication styles complicates the collaborative process, potentially increasing the likelihood of conflict among team members, making the ability to navigate conflict productively a key aspect of communication education. Conflict is an inevitable part of our daily personal and professional lives, and well‐managed conflict can generate positive outcomes, including raising important issues, clarifying individual and team goals,^4^ and helping different parties come to mutual understanding.^5^ However, conflicts may also have negative impacts on the work culture, including increased turnover among health professionals^6^ and negative impacts on the quality of care.^7^, ^8^ While the exact nature of these relationships may be unclear, it has been emphasised that conflict management training represents an important skill for both medical^9^, ^10^ and veterinary^1^, ^2^ professionals.

Conflict can be defined as a situation in which the concerns of two or more people or teams appear to be incompatible. Based on this definition, several researchers have described the ‘dual concerns model of conflict’, which refers to the fact that there are two dimensions of conflict: (1) the extent to which an individual attempts to satisfy one's own concerns (assertiveness) and (2) the extent to which the individual attempts to satisfy the other person's concerns (cooperativeness).^11^, ^12^ Based on these two dimensions, Thomas and Kilmann^13^, ^14^ describe five styles, or modes, of conflict management. These include: avoidance (low assertiveness and low cooperativeness), accommodation (low assertiveness and high cooperativeness), competition (high assertiveness and low cooperativeness), collaboration (high assertiveness and high cooperativeness) and compromise (moderately assertive and cooperative) (Table 1).^13^, ^14^ Based on these, a validated instrument classifying conflict‐handling modes, the Thomas‒Kilmann Conflict Mode Instrument (TKI, Meyers‒Briggs Company) was created.^13^, ^14^ While individuals may adapt or tailor their use of conflict style to the situation, most individuals have a preferred, or dominant, conflict mode. This preferred mode is related in part to personal predispositions, communication competency^15^ and the requirements of the situation. Understanding one's preferred conflict style is important, as this can enhance learners’ self‐awareness and better prepare them to more productively engage in conflict in the future.^16^

**TABLE 1:** Conflict‐handling modes, dimensions, definitions and examples.

Mode	Assertiveness	Cooperativeness	Definition	Examples
Avoiding	Low	Low	Does not address conflict	Sidestepping an issue, postponing issue until a better time
Accommodating	Low	High	Neglects concerns of self to satisfy needs of the other person	Selfless generosity, yielding to another's point of view
Competing	High	Low	Pursues own concerns at other's expense	Standing up for one's rights, defending a position you believe is correct, trying to win
Compromising	Moderate	Moderate	Finding an expedient, mutually acceptable solution that partially satisfies both parties	Seeking a quick middle‐ground position
Collaborating	High	High	Work with other person to find a solution that satisfies concerns of both	Exploring a disagreement to learn from another's insights, finding creative solution to a problem

Multiple factors may influence an individual's approaches to conflict management, including hierarchy and gender, with individuals in positions of lower hierarchy and women tending towards avoiding or accommodating approaches to conflict management.^17^ Research exploring the conflict styles of health professionals has shown that accommodation, avoidance^12^, ^18^, ^19^ and compromising^17^, ^20^ are the most common modes, although this may vary depending on role within the healthcare team^20^ and stage of career.^21^ A recent study of medical students revealed that accommodating and avoiding modes were the most common,^22^ and previous research in veterinary medicine shows similar patterns, with veterinary medical students describing patterns of behaviour consistent with accommodation or avoidance.^16^ Importantly, both accommodation and avoidance styles tend to increase stress in the workplace,^12^ thus creating a suboptimal environment wherein more conflict is likely. In healthcare settings, in particular, the cost of accommodating or avoiding conflict can be particularly costly with respect to medical errors and patient safety.^8^

Despite these preferences towards accommodation, avoidance and compromise, it is possible to adapt one's conflict style,^12^ making education and training related to conflict management, emotional intelligence, communication competence and assertiveness imperative for all health professionals.^1^, ^9^ Prior research related to perceptions of conflict management and conflict management styles supports the positive impact of training on enhanced understanding of conflict and a commitment to collaborative approaches^16^ and shift in predominant conflict styles.^23^, ^24^ While previous research on the self‐reflections of veterinary students has shown that they demonstrate attitudes and behaviour patterns related to conflict avoidance,^16^ no research to‐date has systematically characterised the specific preferred conflict modes of this population using a validated instrument. The purpose of this preliminary pilot study is to characterise first‐year veterinary medical students’ preferred modes of conflict management. Gathering these baseline data will allow for further exploration of how conflict styles develop and change over time and will inform future instruction around effective conflict management.

## MATERIALS AND METHODS

### Participants

Participants were first‐year veterinary students enrolled in a required, 1‐credit team communication course at the North Carolina State College of Veterinary Medicine during the first semester of the Doctor of Veterinary Medicine curriculum in the Fall of 2023. The purpose of this course is to prepare students to collaborate productively as members of a veterinary medical team. Course concepts include, but are not limited to, competent communication, collaboration and coordination strategies, feedback and conflict resolution. One week prior to the course, all students from the class were invited to participate in this study, which was granted exemption by the University Institutional Review Board (#26178). As part of their normal coursework, all students were asked to complete the TKI prior to participation in the course activity. While completion of this survey was considered a mandatory class assignment, students had the opportunity to ‘opt‐in’ or ‘opt‐out’ of having the results from their data be shared for research purposes. The inclusion criterion was first‐year veterinary students who had submitted a signed informed consent form to have their data used for research purposes. The participants received a secure link to complete the survey on the Elevate platform. Following completion of the survey, students received an e‐mail containing their report.

Demographic data were not collected from individual participants to preserve anonymity but were extrapolated from the class of 2027 composite demographic information.

### Survey instrument: TKI

The TKI was used to measure each individual's preferred conflict‐handling mode. This instrument was chosen due to its widespread use in healthcare education and research.^20^, ^21^, ^22^, ^23^, ^25^ The TKI is a validated, proprietary survey consisting of 30 paired statements relating to how individuals prefer to approach conflict. A maximum raw score of 12 can be achieved for each mode. Individual raw scores were compared against the norm frequency to develop percentiles, which was based on a sample of 4000 men and 4000 women, aged 20‒70 years, who were employed full‐time in the United States. These percentile scores were drawn from a database of 59,000 cases collected between 2002 and 2005 and were sampled to ensure representative numbers of people by organisational level and race/ethnicity.^26^ Based on the percentile score, the tendency to use each mode was categorised as ‘high’ (75th percentile and above), ‘medium’ (25th‒74th percentile) or ‘low’ (1st‒25th percentile) for each individual.^11^

### Data analysis

For each participant, a TKI profile was generated by the Myers‒Briggs Company. This profile included information on an individual's preferred conflict‐handling mode, and both a percentile score and a raw score for each conflict‐handling mode for each individual. A single investigator (K.E.M.) who was not involved in the teaching of this course compiled the reports and anonymised and compiled the data for analysis. Descriptive statistics were performed on these data.

## RESULTS

### Participants

One hundred sixteen students participated in the conflict module in the team communication course. Ninety‐one (*n* = 91) students consented to participate in the study, and of these, we received completed surveys from (*n* = 87) students for a 75% response rate.

### Demographic data of the class of 2027

To protect anonymity, we did not gather demographic data from the sample of students who completed the TKI. Instead, we provide the class of 2027 composite demographic information to illustrate that the sample is mostly female (90%), white (66%), and between the ages of 20 and 25 years (82%).

### Preferred conflict‐handling mode for each participant

Accommodating was the most common preferred conflict‐handling mode (*n* = 35/87; 40.2%), followed closely by avoiding (*n* = 29/87; 33.3%). The competing (*n* = 8/87; 9.2%), compromising (*n* = 8/87; 9.2%) and collaborating conflict modes (*n* = 7/87; 8.0%) were the preferred mode in a minority of participants (Table 2).

**TABLE 2:** Preferred conflict‐handling mode.

Conflict‐handling mode	Total (%) (*n* = 87)
Avoiding	29 (33.3)
Accommodating	35 (40.2)
Competing	8 (9.2)
Compromising	8 (9.2)
Collaborating	7 (8.0)

### Number of conflict modes above the 75th percentile

Most participants (*n* = 51; 58.6%) had two or more preferred styles greater than or equal to the 75th percentile (Table 3). Approximately one‐third of participants (*n* = 30, 34.5%) demonstrated one mode above the 75th percentile, and a minority of participants (*n* = 6; 6.9%) did not demonstrate any conflict modes above the 75th percentile. For the participants with two or more preferred styles greater than or equal to the 75th percentile (*n* = 42), the combination of avoiding and accommodating styles was the most common (*n* = 24/42; 57.1%). For the participants with three or more preferred styles (*n* = 9), the most common combination of preferred styles was avoiding, accommodating and compromising (*n* = 3/9; 33.3%).

**TABLE 3:** Total number of conflict‐handling modes greater than or equal to the 75th percentile.

Number of modes ≥75th percentile	Number of participants	Percent of participants	Cumulative percent
0	6	6.9	6.9
1	30	34.5	41.4
2	42	48.3	89.7
3	9	10.3	100.0
Total	87	100.0	

## DISCUSSION

This preliminary study found that first‐year veterinary students expressed a preference for the accommodating conflict‐handling mode, followed by the avoiding conflict‐handling mode. These findings are consistent with previous research, which has identified the accommodating and avoiding styles as common conflict‐handling modes among medical professionals.^12^, ^18^, ^19^, ^22^, ^27^ Some of this may be influenced by stage of training or role. A recent study of fourth‐year medical school students revealed that accommodating was the most common style.^22^ Other studies of healthcare professionals have identified similar tendencies towards accommodation.^12^, ^18^ The accommodating style, which is unassertive and cooperative, focuses on satisfying the other person's needs or concerns over one's own. This might take the form of selfless generosity, obeying another's order when one would prefer not to, or yielding to another person's point of view.^26^ The strong preferences towards the accommodating style in this cohort may be due in part to the ‘student’ mindset that centers on appeasing the other party in a hierarchical situation. Because medical students are often the most junior members of a medical team in a complicated hierarchical system that involves a senior clinician, residents, interns and nurses, the accommodating communication style may be more adaptive for this role. In fact, research has shown that the different communication modes may vary depending on the role within the healthcare team^20^ and stage of career,^21^ suggesting that one's current role may have an impact on an individual's preferred conflict style.

Other studies on the conflict styles of healthcare professionals highlight tendencies towards avoidance.^12^, ^18^, ^19^ The avoiding style is unassertive and uncooperative. When using this style, an individual does not immediately pursue one's own concerns or those of the other person; he or she does not address the conflict. This style may take the form of sidestepping an issue, postponing an issue until a better time, or withdrawing from a threatening situation.^28^ The student mindset may also contribute to the prevalence of the avoidance mode in the current study, as this style may be advantageous when one perceives no chance of satisfying one's concerns, for example, when an individual has low power.^28^ This is relevant because veterinary students operating in a hierarchical medical system often possess the least amount of power on the team. Previous research in veterinary medicine has shown similar results with veterinary medical students describing patterns of behaviour related to accommodation and avoidance.^16^ While the avoiding and accommodating styles may occasionally be adaptive for junior members on a healthcare team, it is important to note that, in general, both accommodation and avoidance styles tend to increase stress in the workplace.^12^ Specifically, individuals with an avoiding style (low assertiveness and low cooperativeness) tend to have a stronger desire to downplay or ignore disputes instead of addressing the issue, which makes it more challenging to generate solutions to workplace conflict.^12^ On the other hand, individuals with an over‐reliance on an accommodating style (low assertiveness and high cooperativeness) may not assert for themselves effectively or advocate for their own interests,^12^ which may result in conflict that does not get addressed. The issue of unaddressed conflict in veterinary practices has real‐life consequences in the work environment. For example, a recent study of veterinarians and registered veterinary technicians in private practices in Canada demonstrated that conflict avoidance or ignoring conflict was one of the top factors negatively impacting team function.^29^

In our study, collaboration was the least common conflict‐handling mode, selected by only 8% (*n* = 7/87) of participants (Table 2). This is consistent with findings of previous research, which has identified collaboration as an uncommon conflict mode among allied healthcare professionals and nurses.^20^ The collaborating style is both assertive and cooperative. When using this style, an individual attempts to work with the other person to find a solution that fully satisfies the concerns of parties. This style might take the form of exploring a disagreement to learn from each other's insights or confronting and trying to find a creative solution to an interpersonal problem.^28^ This process of collaboration in conflict develops solutions that are satisfying to both sides, thus creating optimal and ‘win‒win’ outcomes for both parties.^30^ While previous research has shown that veterinary students frequently acknowledge the importance of collaboration and express a desire to use this conflict mode,^16^ it appears that the collaboration may not be a natural conflict mode for many students. Therefore, it is important for educators to provide training to both enhance students’ understanding of the collaborative mode and increase their efficacy with use of this style during future conflict scenarios. Some examples of this could include didactic training and role‐playing in simulated scenarios with a combination of self‐reflection and real‐time feedback. Previous studies related to conflict management training in healthcare education have shown the potential value of incorporating comprehensive conflict management training into medical education. Specific benefits have been shown, including increases in participants conflict management knowledge, attitudes and skills^31^, ^32^, ^33^ and decreased levels of stress within the workplace.^34^ While these early studies are promising, additional research is required to evaluate the most effective ways to incorporate this instruction into veterinary medical training.

Collaboration, although time‐ and resource‐intensive, offers the advantage of addressing conflict in a manner that may satisfy both parties.^20^ This style may be of particular relevance in the healthcare professions because a junior team member operating in a hierarchical environment may need to advocate for the needs of their patient, rather than giving into the wishes of more senior members of the team.^20^, ^27^, ^35^, ^36^ In a study of health professionals in a university hospital setting, the use of collaboration in conflict scenarios reduced the experience of task and relationship conflict, which reduces an individual's experience of work‐related stress.^12^

It is important to note that there is no ‘best’ conflict style, and effective conflict management involves selection of a style that best fits the circumstances, relationships and individual and professional goals.^20^ Because effective conflict management requires flexibility with a variety of styles depending on the context of the conflict,^20^ we also looked specifically at participants who scored at or above the 75th percentile in more than one conflict style. Over half of participants (Table 3) demonstrated two or more preferred styles greater than or equal to the 75th percentile. These results are similar to the work of previous researchers who studied allied health professionals and nursing students and found that more than half of these participants had two or more conflict styles at or above the 75th percentile.^20^ Further conflict training may increase flexibility with the number of styles, and further longitudinal study would be needed to re‐assess this over time.

Our study has some limitations. To protect the anonymity of participants, we did not collect any direct demographic data from our sample population. Instead, we extrapolated our demographic data based on the college records from the class of 2027 compositive demographic information, which illustrated that the sample was mostly female, white, and between the ages of 20 and 25 years. This is consistent with the broader demographics among veterinary medical students in the United States, which represented by a majority of white, female students attending veterinary school immediately after conferral of their undergraduate degree.^37^ This represents a study limitation because an individual's background, including age, culture, gender and geographic background, may all impact an individual's conflict management preferences.^21^ Our population demographics do not necessarily reflect the ‘normed’ TKI data set of 4000 men and 4000 women of a diverse age range, which may have impacted our results. A second limitation is related to the fact that our results are based on self‐reported data at a single point in time. While self‐reported data related to thoughts and intentions have been shown to be an accurate predictor of future behaviour,^38^ this may not always translate to real‐world behaviour, particularly in high‐stress, real‐world conflict situations. In addition, conflict styles for a single individual are malleable and can change with age and life experience and education.^39^ In this study, our survey was distributed 1 week prior to the course, which could have impacted our results. As students develop a deeper, more nuanced understanding of conflict, their attitudes and attitudes and behaviours in conflict scenarios may shift, which could have influenced their response to the survey questions.

Another study limitation is related to the fact that only a single investigator (K.E.M.) compiled the reports and anonymised the data for analysis, which could have induced errors into the data analysis process. A final limitation is related to the fact that this study was conducted at a single institution, which might affect generalisability to more diverse populations of veterinarians and students across the world. Investigating conflict and communication across the globe is important, especially because of the important interaction of culture and communication on conflict. Specifically, previous research has shown that healthcare professionals from specific ethnic or cultural backgrounds may be more likely to display the accommodating style than other racial or ethnic cohorts, potentially due to differences in culture and socialisation.^21^ This represents an area for future study. Future research in this area may also focus on exploring how the conflict styles of veterinary students may change over time as they progress through their training and professional socialisation and on the application of conflict preferences to real‐world medical settings.

This study further highlights the need for additional educational opportunities on the topic of conflict resolution. Providing training and active learning opportunities related to conflict communication and flexibility with conflict management styles may be a method to engage in conflict more productively. Instruction on a variety of conflict styles may lead to increased comfort and flexibility among students in selecting the appropriate conflict mode to meet the needs of the individual conflict scenario.^40^ In addition to educational interventions, preferred conflict styles can shift over time as individuals become more comfortable with assertiveness and cooperativeness. In other words, education and training, coupled with opportunities to engage in conflict more productively, can result in a shift away from accommodation and avoidance towards more collaborative approaches to conflict resolution.^31^

In closing, our current study found that most students expressed a conflict preference for accommodating, followed by the avoiding conflict style. A minority of students selected collaboration as their preferred conflict style. These findings are important because understanding students’ baseline conflict preferences at the beginning of their veterinary education will help to inform future instruction and curriculum design in conflict resolution. This preliminary pilot study also provides a baseline from which to understand how these conflict preferences may change over time.

## AUTHOR CONTRIBUTIONS

Katherine E. McCool and April A. Kedrowicz planned and designed the research study. Katherine E. McCool led the data gathering analysis and reporting. Katherine E. McCool and April A. Kedrowicz wrote the introduction and discussion and edited the manuscript.

## CONFLICTS OF INTEREST

The authors declare they have no conflicts of interest.

## ETHICS STATEMENT

The authors confirm that that the ethical policies of the journal, as noted on the journal's author guidleines page, have been adhered to. The research was granted exempt status by the Insitirutional Review Board of NC State University.

## Data Availability

The data that support the findings of this study are available upon request from the corresponding author. The data are not publicly available due to privacy or ethical restrictions.
